# Clinicopathological and Dermoscopic Features in Blashkoid Angioma Serpiginosum

**DOI:** 10.5826/dpc.1101a107

**Published:** 2021-01-29

**Authors:** Aditi Dhanta, Gargi Taneja, Neirita Hazarika, Prashant Joshi

**Affiliations:** 1 Department of Dermatology, All India Institute of Medical Sciences, Rishikesh, India; 2 Department of Pathology, All India Institute of Medical Sciences, Rishikesh, India

**Keywords:** punctate macules, angioma, lagoon, dermoscopy

## Introduction

Angioma serpiginosum (AS) is an unusual vascular nevoid disorder that is more common in women. It begins in childhood and stabilizes in adulthood. Dermoscopy of angioma serpiginosum reveals typical small, sharply demarcated, round-to-oval red lagoons, corresponding histopathologically to dilated, thin-walled capillaries found in the superficial dermis.

## Clinical Presentation

**Figure 1:**
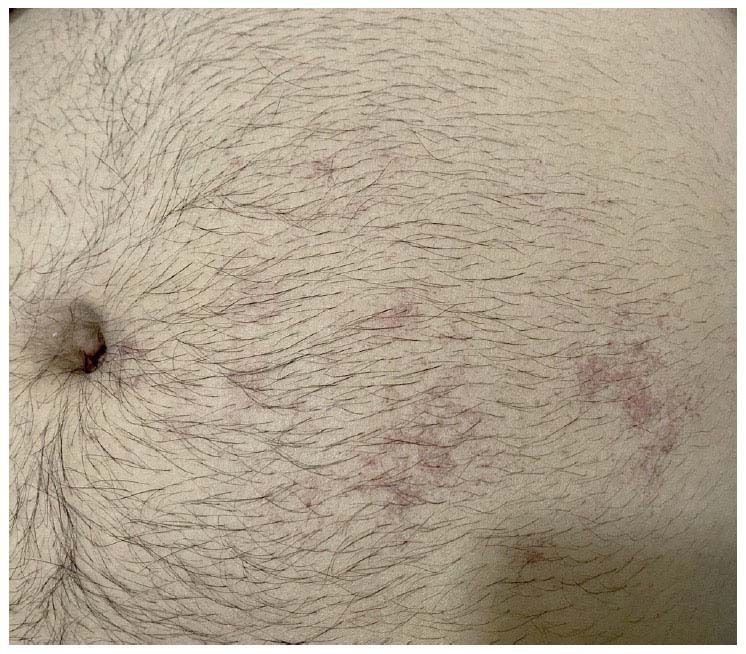
Multiple pinpoint, grouped, bright red, nonblanchable macules and irregular patches distributed in a patchy blaschkoid pattern over the left side of abdomen.

A 29-year-old man presented with a 10-year history of asymptomatic red lesions over the left side of abdomen. There was no associated history of itching or trauma. General and systemic examination was normal. Cutaneous examination revealed multiple minute, pinpoint, grouped, bright red, nonblanchable macules and irregular patches distributed in a patchy blaschkoid pattern over the left side of abdomen (Figure 1).

**Figure 2:**
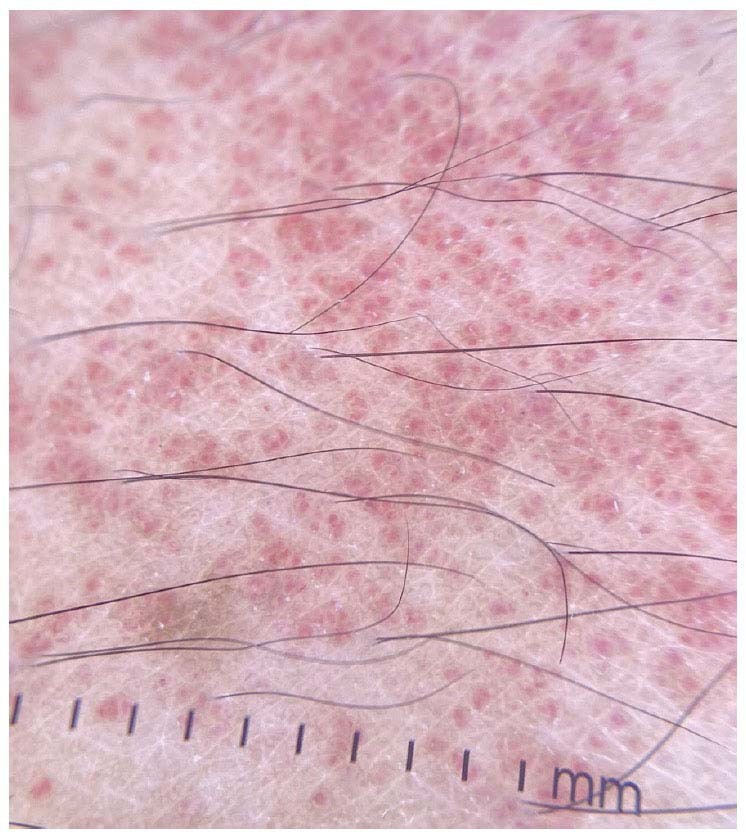
Dermoscopy of the erythematous lesions using a Derm-Lite II Hybrid M Dermatoscope, polarized mode, ×10 magnification, revealed multiple oval-to-round, well-demarcated, red-colored lagoons present on a slightly erythematous background.

Dermoscopy of the erythematous lesions using a 3Gen DermLite II Hybrid M dermatoscope, polarized mode, ×10 magnification, revealed multiple oval-to-round, well-demarcated, red-colored lagoons on a slightly erythematous background (Figure 2). Lesions were nonblanchable on diascopy.

**Figure 3:**
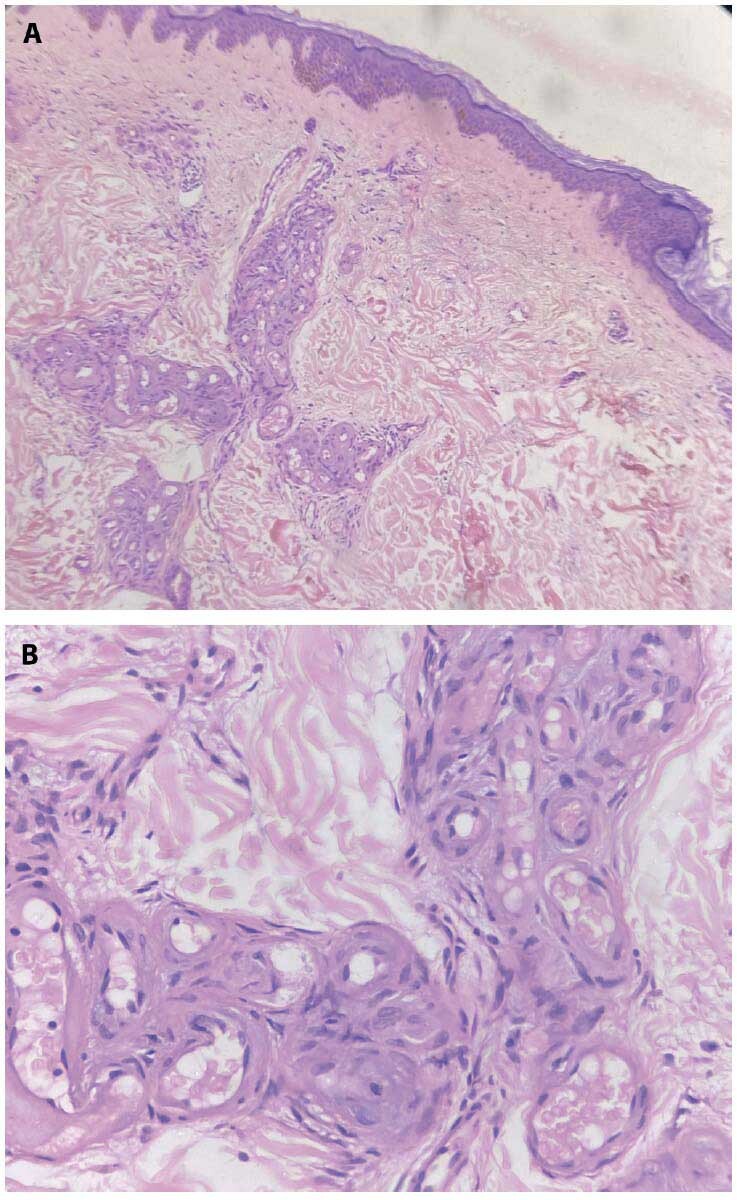
(A) Photomicrograph showing multiple dilated vessels present in a clover-leaf pattern in the upper dermis (H&E, ×10). (B) Photomicrograph showing cluster of dilated thick-walled capillaries (due to deposition of PAS-positive material) with flattened endothelial cells and red blood cells present in the center of the vessels (H&E, ×40).

A punch biopsy of 4 mm taken from erythematous lesions revealed the presence of mild hyalinization and increased proliferating small capillaries with a normal endothelial lining in the papillary and superficial reticular dermis (Figure 3). There were neither epidermal changes nor extravasation of red blood cells.

## Conclusions

AS is an uncommon benign, vascular nevoid disorder first described by Hutchinson in 1889 [1]. The exact etiopathogenesis is unknown; however, a hyperestrogenic state is considered to play role in pathogenesis owing to its proliferative effects on vascular endothelial cells. A recently proposed etiology is an abnormal vascular response to cold that manifests as formation and aggregation of newly formed capillaries that leads to large ecstatic vessels in the papillary dermis [2].

AS typically begins in childhood and has preponderance in women. It is characterized by multiple small, asymptomatic, nonpalpable, deep red punctate macules organized in serpiginous and gyrate patterns. The lesions are predominantly distributed on the lower extremities and buttocks and usually appear unilateral, but bilateral asymmetric involvement or linear morphology has been reported, though rarely. Segmental patterns of lesions, as seen in our case, may reflect cutaneous mosaicism.

Differential diagnosis includes pigmented purpuric dermatosis, unilateral nevoid telangiectasia, port-wine stain, and angiokeratoma corporis diffusum. Apart from the clinical presentation, confirmation of diagnosis is made by histopathology. A characteristic histopathological feature of AS is the vascular proliferation located at the papillary dermis, which is composed of dilated capillaries [2]. The lack of epidermal changes and extravasation of red blood cells distinguish AS from angiokeratoma and pigmented purpuric dermatosis.

Clinical findings alone or a noncontributory biopsy report can be misleading, so dermoscopic features help to distinguish AS from other vascular-related diseases. The characteristic dermoscopic findings reported in the literature include erythematous, well-demarcated, round-to-oval dots and lagoons [1,2]. The red lagoons represent dilated vascular spaces within the papillary or superficial reticular dermis. Table 1 lists common differentials of AS and their dermoscopic features.

AS is a benign disease and commonly asymptomatic requiring no treatment, although it raises cosmetic issues. There are several reports describing intense pulsed light or pulsed dye laser as treatment, with a clearance rate ranging from 25% to 75% [2].

## Figures and Tables

**Table 1:** Dermoscopic Findings of Differential Diagnosis of Angioma Serpiginosum

Differential Diagnosis	Dermoscopic Features
Angiokeratoma corporis diffusum	Dark lacunae Blue-whitish veil Ulceration Rainbow pattern [3]
Pigmented purpuric dermatosis	Coppery-red background Round-to-oval dots Gray dots Network [3]
Port-wine stain	Deep form—red linear vessels and represent horizontally oriented capillaries Superficial form—red, rounded, globular vessels [3]
Unilateral nevoid telangiectasia	Dense network of linear, tortuous. and branching telangiectasia [1]
